# Use of daily electronic patient-reported outcome (PRO) diaries in randomized controlled trials for rheumatoid arthritis: rationale and implementation

**DOI:** 10.1186/s13063-019-3272-0

**Published:** 2019-03-22

**Authors:** Clifton O. Bingham, Carol L. Gaich, Amy M. DeLozier, Kathryn D. Engstrom, April N. Naegeli, Stephanie de Bono, Pixy Banerjee, Peter C. Taylor

**Affiliations:** 1 Divisions of Rheumatology and Allergy and Clinical Immunology, Johns Hopkins University, 5200 Eastern Avenue, MFL Center Tower Room 404, Baltimore, MD 21224 USA; 2 Eli Lilly and Company, Indianapolis, USA; 3 Eli Lilly Services India Private Limited, Bangalore, India; 4 Botnar Research Centre, Nuffield Department of Orthopaedics, Rheumatology and Musculoskeletal Sciences, University of Oxford, Windmill Road Headington, Oxford, UK

**Keywords:** Daily electronic diary, ePRO, Patient-reported outcome, Rheumatoid arthritis

## Abstract

**Background:**

Rheumatoid arthritis (RA) is associated with significantly diminished health-related quality of life. Patient-reported outcomes (PROs) are considered important in RA; however, some symptoms such as morning joint stiffness (MJS) and fatigue that are considered important by patients are not captured by the American College of Rheumatology “core set” measures for RA trials. The US Food and Drug Administration has endorsed electronic capture of clinical trial data including PROs, and electronic PRO (ePRO) systems may lead to more accurate and complete data capture, improved compliance, and patient acceptance compared with paper-based methods. Our objective was to assess the implementation of ePRO measures of Duration and Severity of MJS, Severity of Worst Tiredness, and Severity of Worst Joint Pain in baricitinib RA-BEAM and RA-BUILD phase 3 randomized clinical trials (RCTs).

**Methods:**

A daily electronic diary (handheld device; Invivodata®, Inc.) was utilized to capture PRO data in the RCTs. Three “reporting window” options were incorporated to accommodate differences in patients’ routine daily schedules, and alarms were programmed for each reporting window. Duration of MJS was recorded in “hours and minutes,” and Severity of MJS, Worst Tiredness, and Worst Joint Pain were captured on a 0 to 10 rating scale, with a higher score indicating more severe symptoms. The patients and site staff were trained to use the daily electronic diary.

**Results:**

Patients with moderately to severely active RA used the daily electronic diary in the RA-BEAM study (*N* = 1305) and RA-BUILD study (*N* = 684). The average compliance, calculated as total days completed by patients compared with total days expected to complete the diary, through Week 12 was high (RA-BEAM 94% patients; RA-BUILD 93% patients), potentially attributable to appropriate training, clarity of instructions, simple user interface, and electronic device design. Identified process challenges included non-timely issuance of the device, low battery, inadequate training of patients before data collection, inappropriate diary set-up, and first response entry 1 day after the baseline visit.

**Conclusions:**

High compliance rates support the use of the daily electronic PRO diary in large RCTs. Despite the anticipated issues, the daily electronic diary is expected to reduce recall bias and improve the quality of PRO data collection.

**Trial registration:**

RA-BEAM (NCT01710358) and RA-BUILD (NCT01721057).

## Background

Rheumatoid arthritis (RA) is a systemic, inflammatory, autoimmune disease. RA has variable expression and outcomes, ranging from mild to severe, and is associated with progressive joint destruction, significantly compromised health-related quality of life (HRQoL), and reduced survival [1, 2]. Clinical trials assess the effect of drugs on certain aspects of RA, including symptoms, structural joint damage, and biomarkers of inflammation [3]. However, assessments undertaken by physicians in a clinical trial may not adequately capture the impact of disease from a patient’s perspective [3, 4]. Symptoms and impacts of the disease are best known by the patient and therefore best measured by patient-reported outcomes (PROs) [3]. The Food and Drug Administration (FDA) guidance for industry recognizes that PRO instruments should be used in measuring concepts best known by the patient or best measured from a patient’s perspective in clinical trials [5]. There has been a growing interest in the use of PROs in RA [6, 7]. Patients with RA have identified pain, physical function, fatigue, joint stiffness, participation, sleep, and emotional and psychosocial factors as important domains for the assessment of HRQoL [8–10].

Traditionally, in randomized clinical trials (RCTs) and clinical care, PRO measures have been administered to patients in paper format during scheduled visits or face-to-face encounters [11, 12]. Paper and electronic diaries have been used in studies to capture patient-reported events between visits, more proximate to their occurrence [13]. However, paper-based methods have the risk of participants completing multiple days of reporting all at once, referred to as the “parking lot effect,” in which all responses may be completed just before the visit while sitting in the parking lot. Thus, there is an increasing use of electronic PRO (ePRO) systems to gather PROs for more accurate and complete data capture, improved compliance, decreased likelihood of data entry errors, reduced administrative burden, and higher acceptance by respondents [11]. Most ePRO systems provide date and time stamps for each data entry and restrict data entry to specific periods, thereby avoiding backward or forward filling of entries. ePRO systems can also reduce missing or unusable data by ensuring data capture at the right time [11]. Additionally, there is a potential for diminished accuracy of reporting with respect to recall of more distant events, which exists with all instruments [13]; daily assessments of PROs may capture patient experiences more accurately by minimizing recall bias.

With the increased use of electronic systems to improve documentation of source data in clinical trials, the FDA has endorsed electronic capture of clinical trial source data, including PRO endpoints [14], and has preferred the electronic form over paper-based data collection [11]. The FDA has developed the guidance for industry on the use of computerized systems for clinical investigations [14]. The FDA has also recognized that source data captured electronically should be attributable, legible, contemporaneous, original, and accurate (ALCOA) to ensure data quality and integrity, and must meet regulatory requirements for recordkeeping [15]. To ensure appropriate implementation of ePRO systems, several recommendations have been published by the International Society for Pharmacoeconomics and Outcomes Research (ISPOR) [11, 16–19].

Electronic PRO data collection in clinical trials can be conducted on site (clinic) or off-site (patient’s home, workplace, or school); the off-site assessment is considered to be optimal, as it allows for accurate and real-time reporting of symptoms by patients. Handheld touchscreen-based devices have become the mainstay for unsupervised, off-site PRO data collection, especially for clinical trials requiring frequent data entry [11]. ePRO data collection has been utilized in RA RCTs to record pain, disability, and tender joint counts during patients’ study visits and is preferable to patients compared to paper-based measurement [20].

Although patients have identified morning joint stiffness (MJS) and fatigue as important disease-related symptoms [21–24], none of these are included in the composite disease activity indices, such as Disease Activity Score 28 (DAS 28), Clinical Disease Activity Index (CDAI), and Simplified Disease Activity Index (SDAI), which are used in RA clinical trials. As part of a recent clinical development program of baricitinib for the treatment of RA, PRO measures reflecting Duration and Severity of MJS, Severity of Worst Tiredness, and Severity of Worst Joint Pain were developed and incorporated into the RA-BEAM (NCT01710358) and RA-BUILD (NCT01721057) phase 3 RCTs [25, 26], considering the importance of PROs in RA. A handheld ePRO device was used to capture these measures daily. This manuscript summarizes the rationale for the use of the daily ePRO electronic diary, its implementation in the baricitinib phase 3 studies, and real-time patient compliance to the diary.

## Methods

### Components and function of daily electronic diary

**Fig. 1:**
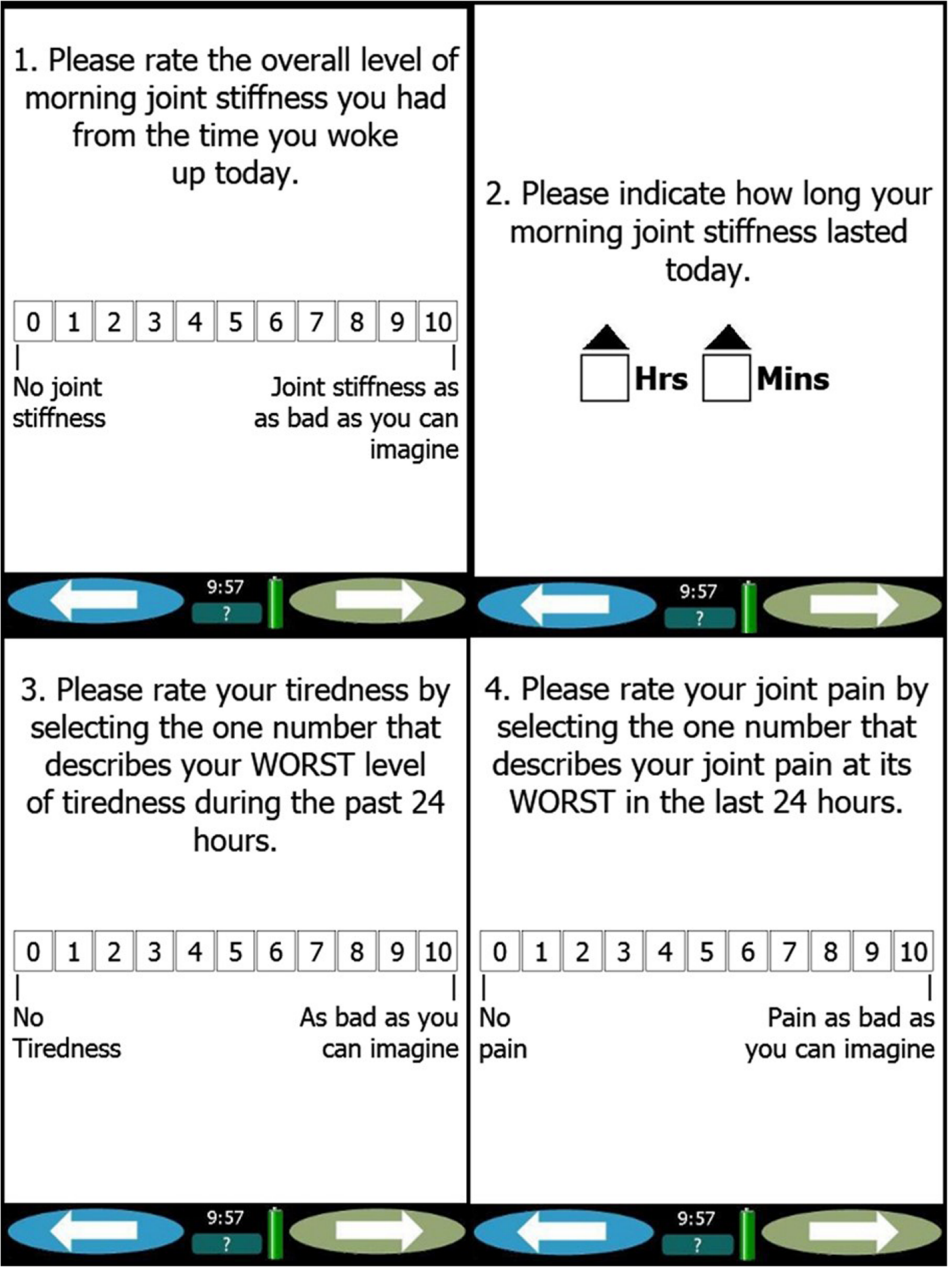
Questions to evaluate the four gated measures through daily electronic diary

The daily electronic diary, a device provided by Invivodata®, Inc., is a self-administered PRO assessment tool that was used to assess MJS, tiredness, and joint pain in the two clinical trials of baricitinib [25, 26] (Fig. 1). As reported in previous publications, the content validity and psychometric properties of the MJS and Worst Tiredness have been assessed, utilizing the data from the RA-BEAM and RA-BUILD studies [27, 28].

The ePRO information was collected in accordance with the PRO guidance from the FDA, with data collected in the daily diary using a handheld device provided to patients. Daily assessments were recorded beginning with the end of the patient’s day of the baseline visit (Visit 2, Day 1) and were continued on subsequent days through Week 12. The primary analyses in both trials were based on scores collected in the 7 days prior to the Week 12 visit date.

Diary entries could be made only during the 5-h 45 min “reporting window” chosen during diary set-up at the site. Three “reporting window” options were incorporated to accommodate differences in patients’ routine schedules (Table 1). The electronic diary allowed patients to enter the data within the selected reporting time window at the end of the patients’ day. Alarms were also programmed into the device based on the selected window to remind patients to complete the electronic diary.

**Table 1:** PRO daily electronic diary reporting windows and alarm alerts

	Daily report window	Electronic diary PRO alarms^a^
Standard	6:00 p.m. – 11:45 p.m.	8:00 p.m., 9:00 p.m., 10:00 p.m., and 11:00 p.m.
Shift worker A	6:00 a.m. – 11:45 a.m.	8:00 a.m., 9:00 a.m., 10:00 a.m., and 11:00 a.m.
Shift worker B	12:00 p.m. – 5:45 p.m.	2:00 p.m., 3:00 p.m., 4:00 p.m., and 5:00 p.m.

^a^The patient could complete the daily report as early as their window allowed and did not need to wait until the electronic diary alarmed to complete the daily report. Once the patient completed the daily report, the daily report button disappeared from the electronic diary screen, and all remaining alarms were canceled

The electronic diary utilized a built-in cellular card to transfer data wirelessly to a central server, the EPX web-based system, which allowed data transfer without the use of an Internet connection. The data transfers occurred automatically overnight in the local time zone if the device was adequately charged. The devices displayed a warning message when they were low on charge; the message was received if the patient was interacting using the device and it was not completely discharged. Furthermore, monitoring reports were generated which alerted the sites through an email if patients missed diary entries for 3 or more consecutive days, so the site could follow up with the patient. The monitoring reports were implemented approximately 6 months after the start of the study.

While the treatment blind was maintained in a separate system, the data collected on the daily electronic diary could be viewed by the sites, Clinical Research Associates (CRAs), and sponsors through their access to the EPX website using their unique Login name and password. Sites used the EPX website to manage patients’ data, monitor PRO compliance, and view and print reports. A replacement electronic diary was issued to a patient who had undergone prior training if the electronic diary was lost, damaged, or was not functioning properly. Data collected on malfunctioning devices were extracted and transferred to the web portal.

### Training of patients and site staff

To ensure successful implementation of daily electronic diaries, patients and site staff underwent training in accordance with a training protocol that was employed across all study sites prior to study execution.

Standardized training sessions were conducted for investigators, study coordinators, and designated site staff prior to administration and included five topics: “how to set up the electronic diary for the patient and select a response time window”; “how to train patients to use the electronic diary”; “how to record patient status in the electronic diary”; “how to complete data transfers during site visits”; and “how to monitor patient compliance.”

The site staff underwent face-to-face training and hands-on experience with the devices at investigator meetings or online training modules. Subsequently, site staff provided standardized training to the patients to ensure comprehension and performance using the electronic diary entries. Patients were instructed to charge the electronic diary every evening. The option of using an oversized stylus for completion of the electronic diary was provided to help patients with limited hand function interact more easily with the touch-screen surface. Training was provided on the use of the stylus.

Training was completed for each individual patient, and required 2 h to complete on an average. After the device was set up for a patient, the site instructions were to train the patient, and the device directly entered training mode to minimize the chance of sites forgetting to train the patient. The training mode included a Standard Practice Assessment that each patient completed before the site confirmed “Training Complete” in the device. The training mode had to be completed prior to going into “active” mode. The site user manual also included an instruction to “Review the information on each page of the DiaryPRO Subject Quick Start Guide with your Patient.” The patient was also provided the Subject Quick Start Guide for reference.

Both clinical trials utilizing the electronic diaries were global and included 24/7 telephone support in English in addition to translator support for investigators, site coordinators, and site staff in Chinese, French, German, Spanish, and Japanese during local business hours.

### PRO questions and response options

Daily assessments were conducted to capture the duration of MJS, which was recorded in “hours and minutes” for the length of time that the stiffness lasted each day (Fig. 1). The electronic device showed response options using a scrolling display, with an up arrow and a down arrow to show an increase or a decrease in the selection of the exact duration of time. The arrows allowed ease in scrolling separately through hours and minutes, considering that it could be difficult for the patients to scroll it up for a long time as the duration of MJS usually lasts for more than 1 h. Patients also rated the severity of their MJS, worst tiredness, and worst joint pain for the day on an 11-point (0 to 10) numeric rating scale (NRS), with a score of 10 indicating symptoms “as bad as you can imagine” and a score of 0 indicating no symptoms (Fig. 1). Entries could be recorded by touch with a knuckle, finger, or an oversized stylus to select their level of given symptom.

### Compliance/missed entries

A date and time stamp was attached to collected data. The “time stamping” and reporting “time window” options for entering data into electronic diaries eliminated backward and/or forward diary filling.

Multiple factors can lead to missed entries; thus, alarms and reminders (Table 1) were used to improve compliance with self-reported data. Alarms reminded patients to complete their electronic diaries. Four programmed alarms were individualized, based on the time window, which were mostly aligned to a patient’s end of day. The selected window remained consistent for diary entry of the health outcomes measured by the patient through the first 12 weeks of the studies. An automatic alarm would sound if the electronic diary had not been completed for the day. The devices were programmed to sound alarms during the last 2–4 h of the 5-h 45 min entry window (Table 1).

Missing data were handled according to the statistical analysis plan reported in previous publications [25, 26]. Reasons for missing baseline diary data were determined via review of the diary device log files for RA-BEAM and RA-BUILD trials.

The overall compliance, defined as total days completed by patients compared to the total days patients were expected to complete the daily electronic diary, was assessed through Week 12 in both trials.

### Translation and adaptation of the diary

The translation and cultural adaptations of the electronic diaries were performed using the ISPOR guidelines [29]. This included two independent forward translations, harmonized translations, cognitive debriefing with five patients with RA, desktop publishing of the validated translation, and proofreading of the validated translation for each language and country.

Patients with RA who were native speakers in the target countries reviewed the harmonized translations. Translated documents were debriefed by a trained bilingual interviewer, who recorded patients’ abilities to comprehend the items and made note of their comments. Patients’ comments and comprehension of the translated items were recorded.

## Results and discussion

The daily electronic PRO diary was used by patients with moderately to severely active RA, who were naïve to biologic treatment, and enrolled in the RA-BUILD study (*N* = 684) and the RA-BEAM study (*N* = 1305). The results of PRO assessments using the electronic diary in patients with moderately to severely active RA have been previously reported [25, 26], and the criteria for sufficient reliability, validity, responsiveness, and interpretation standards have been fulfilled. Prior qualitative studies concluded that the diary instructions were clear, items were relevant, and the devices were easy to use [27, 28].

The overall compliance with daily electronic diary reporting through Week 12 was high (more than 90%) in both the RCTs. Overall, the compliance rates were observed to be similar for both the trials: 94% in the RA-BEAM trial and 93% in the RA-BUILD trial.

The percentage of patients completing the daily electronic diary was also more than 90% throughout Week 1 to Week 12 for both the trials. The percentage of patients with missing data was high at Week 1 compared to other weeks, which may be attributable to improper device set-up and/or timing of initial data collection (Table 2). The average number of missing days per week was reported to be low in both trials (Table 3). As observed for the percentage of patients with missing data, the average number of days of missing data was also high in Week 1 compared with the other weeks. No specific pattern was observed for missing data across the remaining weeks.

**Table 2:** Percentage of patients completing the responses by visit/week

Study ID	Wk 1	Wk 2	Wk 3	Wk 4	Wk 5	Wk 6	Wk 7	Wk 8	Wk 9	Wk 10	Wk 11	Wk 12
RA-BEAM	92.7	97.0	97.5	97.2	98.2	98.2	97.9	97.3	97.9	97.6	97.3	93.7
RA-BUILD	91.7	94.9	97.0	95.8	97.3	97.4	96.4	96.5	97.0	95.3	95.3	91.9
Overall	92.4	96.2	97.3	96.7	97.9	97.9	97.4	97.0	97.6	96.8	96.6	93.1

*Abbreviation*: *Wk* Week

**Table 3:** Average number of missing days for each visit

Study ID	Wk 1	Wk 2	Wk 3	Wk 4	Wk 5	Wk 6	Wk 7	Wk 8	Wk 9	Wk 10	Wk 11	Wk 12
RA-BEAM	0.71	0.34	0.30	0.32	0.28	0.27	0.29	0.37	0.31	0.33	0.37	0.97
RA-BUILD	0.74	0.50	0.37	0.43	0.35	0.33	0.42	0.43	0.41	0.49	0.56	1.04
Overall	0.72	0.39	0.32	0.36	0.30	0.30	0.33	0.39	0.34	0.38	0.44	0.99

*Abbreviation*: *Wk* Week

Several reasons were noted by the study team for missing diary data at baseline (day of first dose) (Table 4). First, if the preferred reporting window closed prior to baseline device set-up, then the window would not reopen until the next day. If the preferred reporting window was 6:00 a.m. – 11:45 a.m. or 12:00 p.m. – 5:45 p.m. and the patient’s baseline visit occurred during or after the reporting window, the first reminder alarm would not sound until the reporting window for the next day. Second, the users abandoned or delayed the report past the reporting window. Third, patients could have ignored the device alarms or left the device out of hearing range; however, a record of the missed alarms was captured in the log file. Fourth, the sites were required to provide patients with a fully charged device; however, low battery levels (battery level ≤ 5%) were detected at the baseline visit date in some cases. This may have interfered with a patient’s ability to answer the daily diary report. Fifth, a few site errors were also noted, including delay or failure to provide the device to the patient at the baseline visit. In both the RA-BEAM and RA-BUILD trials, “first alarm next day” was the most common reason (19.6% in the RA-BEAM trial and 30.4% in the RA-BUILD trial) for missing baseline diary data.

**Table 4:** Reasons for missing baseline diary data

Reason	RA-BEAM (*N* = 1307)	RA-BUILD (*N* = 684)
Device never given to patient	17	12
Device given to patient after baseline	130	57
Missed alarms	97	48
Low battery	82	25
First alarm next day	256	208
User abandoned or delayed report past window	171	21
Other	15	1

During the course of this program, the study team learned that there should be a more robust process for tracking lost or defective devices. However, based on the high post-baseline compliance rate, we can surmise that lost or defective devices had minimal impact on data completeness.

Estimates of compliance with paper diaries have been reported to be high in prior studies [30]. However, these estimates have been derived from unconfirmed recordings of diary completion by patients, which might not be accurate. Stone and colleagues compared patient compliance with a paper diary instrumented to track diary use versus an electronic diary in patients with chronic pain [30]. Paper diaries were reported to demonstrate high overall compliance, but the per protocol or actual compliance was very low (11%), which was possibly attributable to the “parking lot effect” [30]. Compliance with the electronic system was reported to be high (94%), which was similar to the findings of our analysis. As reported by previous studies [31, 32], appropriate training, clarity of instructions, a simple user interface, and design of the electronic device to assure protocol compliance might be potential factors contributing to the high compliance rate.

Although the daily electronic diary was effective in collecting data in the two clinical trials, and high compliance was observed, several technical and process issues were identified that should be considered when implementing daily electronic diaries in future studies. These include technical issues with the electronic system (e.g., battery charging) that could limit the utility of electronic diaries in some circumstances, provision of devices in a timely fashion to study subjects, inadequate patient training, improper device set-up, and timing of initial data collection. A comparison of compliance rates between trial arms and between demographic groups was not assessed in this study but should be considered for potential future work. The potential for high compliance rate related to researcher follow-up when the diary was not completed for 3 days could also be explored in future studies.

Notwithstanding the above considerations, daily electronic diaries would be expected to reduce recall bias and enhance the sensitivity to detect changes in PRO data, resulting in reduced error variance [11]. Results from this study suggest that daily electronic diary capture is feasible in large multinational RCTs and further support the use of this method of PRO collection.

## Conclusions

The implementation of daily electronic diaries in the two phase 3 RCTs in patients with RA and the high compliance rates reported by the patients enrolled in these trials support the PRO data collection using daily electronic diaries. Opportunities to employ PROs and diary technology may enhance the monitoring of quality of life for people with RA between face-to-face study visits and clinical encounters. Capturing and reporting more reliable PRO data, collected in real time, can provide further evidence of beneficial treatment effects on outcomes that matter most to patients. Even though RCT sponsors and investigative sites can anticipate certain technology challenges and training needs in deploying electronic diaries, our experiences reported here and in previously reported studies [27, 28] indicate the feasibility, acceptability, and quality of PRO results using this method.

## Abbreviations

ALCOA: Attributable, legible, contemporaneous, original, and accurate
CDAI: Clinical Disease Acivity Index
CRA: Clinical Research Associate
DAS28: Disease Activity 28
ePRO: electronic patient-repored outcome
FDA: Food and Drug Administration
HRQoL: Health-related quality of life
ISPOR: International Society for Pharmacoeconomics and Outcomes Research
MJS: Morning joint stiffness
NRS: Numeric rating scale
PRO: Patient-reported outcome
RA: Rheumatoid arthritis
RCT: Randomized controlled trial
SDAI: Simplified Disease Activity Index
