# Correction to: Use of daily electronic patient-reported outcome (PRO) diaries in randomized controlled trials for rheumatoid arthritis: rationale and implementation

**DOI:** 10.1186/s13063-019-3463-8

**Published:** 2019-06-04

**Authors:** Clifton O. Bingham, Carol L. Gaich, Amy M. DeLozier, Kathryn D. Engstrom, April N. Naegeli, Stephanie de Bono, Pixy Banerjee, Peter C. Taylor

**Affiliations:** 1 Divisions of Rheumatology and Allergy and Clinical Immunology, Johns, Hopkins University, 5200 Eastern Avenue, MFL Center Tower Room 404, Baltimore, MD 21224 USA; 2 Eli Lilly and Company, Indianapolis, USA; 3 Eli Lilly Services India Private Limited, Bangalore, India; 4 Botnar Research Centre, Nuffield Department of Orthopaedics, Rheumatology and Musculoskeletal Sciences, University of Oxford, Windmill Road Headington, Oxford, UK

**Correction to: Trials**

**https://doi.org/10.1186/s13063-019-3272-0**

After publication of the original article [1], the authors have notified us of an error in language used in the text:

- The first sentence of the 6th paragraph under the *Methods* sections should read “Training was completed for each individual patient.”
- The first sentence of the 3rd paragraph under *Training of patients and site staff* should read: “The site staff underwent face-to-face training and hands-on experience with the devices at investigator meetings or online training modules and **took 2 hours to complete on average**.”

The phrase “took 2 hours to complete on average” was moved from the first sentence to the second one.
